# Absent in melanoma 2: a potent suppressor of retinal pigment epithelial-mesenchymal transition and experimental proliferative vitreoretinopathy

**DOI:** 10.1038/s41419-025-07367-9

**Published:** 2025-01-27

**Authors:** Yu Chen, Mingyuan Jiang, Liping Li, Shanshan Yang, Zuimeng Liu, Shiwen Lin, Wanxiao Wang, Jinyang Li, Feng Chen, Qiang Hou, Xiaoyin Ma, Ling Hou

**Affiliations:** 1 Laboratory of Developmental Cell Biology and Disease, State Key Laboratory of Ophthalmology, Optometry and Visual Science, Eye Hospital, Wenzhou Medical University, Wenzhou, 325027 China; 2 Institute of Developmental and Genetic Ophthalmology, Wenzhou Medical University, Wenzhou, 325027 China; 3 Department of Ophthalmology, the Second Affiliated Hospital of Army Medical University, Chongqing, China; 4 Zhengzhou Aier Eye Hospital, Zhengzhou, China; 5 National Clinical Research Center for Ocular Diseases, Eye Hospital, Wenzhou Medical University, Wenzhou, China; 6 State Key Laboratory of Ophthalmology, Optometry and Visual Science, Eye Hospital, Wenzhou Medical University, Wenzhou, 325027 China

**Keywords:** Diseases, Cell biology

## Abstract

Epithelial-to-mesenchymal transition (EMT) is a critical and complex process involved in normal embryonic development, tissue regeneration, and tumor progression. It also contributes to retinal diseases, such as age-related macular degeneration (AMD) and proliferative vitreoretinopathy (PVR). Although absent in melanoma 2 (AIM2) has been linked to inflammatory disorders, autoimmune diseases, and cancers, its role in the EMT of the retinal pigment epithelium (RPE-EMT) and retinal diseases remains unclear. The present study demonstrated that AIM2 functions as a potent suppressor of RPE cell proliferation and EMT to maintain retinal homeostasis. Transcriptome analysis using RNA-sequencing (RNA-Seq) revealed that AIM2 was significantly downregulated in primary human RPE (phRPE) cells undergoing EMT and proliferation. Consequently, *Aim2*-deficient mice showed morphological changes and increased FN expression in RPE cells under physiological conditions, whereas AIM2 overexpression in phRPE cells inhibited EMT. In a retinal detachment-induced PVR mouse model, AIM2 deficiency promotes RPE-EMT, resulting in severe experimental PVR. Clinical samples further confirmed the downregulation of *AIM2* in the PVR membranes from patients. Kyoto Encyclopedia of Genes and Genome analysis revealed that the PI3K-AKT signaling pathway was significantly related to RPE-EMT and that AIM2 inhibited AKT activation in RPE cells by reducing its phosphorylation. Moreover, treatment with eye drops containing an AKT inhibitor alleviated RPE-EMT and the severity of experimental PVR. These findings provide new insights into the complex mechanisms underlying RPE-EMT and PVR pathogenesis, with implications for rational strategies for potential therapeutic applications in PVR by targeting RPE-EMT.

## Introduction

Epithelial-to-mesenchymal transition (EMT) is a fundamental cellular process that involves the loss of epithelial properties and the acquisition of mesenchymal properties in epithelial cells [1]. As EMT significantly affects cell motility and proliferation and modulates embryonic stem cell differentiation [1], this process has been extensively studied in the context of embryonic development [2], carcinoma progression [3], and tissue pathology [4]. The retinal pigment epithelium (RPE) is a monolayer of epithelial cells located between the neural retina and choroid that supports neural retinal function by participating in the visual cycle, secreting neurotrophic factors, clearing reactive oxygen species (ROS), maintaining the blood-retina barrier, and removing retinal waste to support retinal homeostasis [5, 6]. Under normal conditions, mature RPE cells remain quiescent throughout life [7]. However, increasing evidence suggests that RPE cells undergo EMT and exhibit enhanced migration in various retinal diseases, such as proliferative vitreoretinopathy (PVR) [8] and age-related macular degeneration (AMD) [9, 10].

PVR is a common complication in 40–60% of patients with open-globe injuries and 5–10% of patients with retinal detachment (RD) [11, 12]. Under these conditions, when injury to the retina occurs, RPE cells detach from Bruch’s membrane, undergo EMT and proliferation, and migrate into the vitreous through tears of the neural retina, thereby contributing to the formation of the epiretinal membranes. Eventually, the formation of PVR membranes leads to tractional RD and visual impairment. RPE-EMT is a hallmark of PVR pathogenesis [12, 13].

The myofibroblast transdifferentiation of RPE cells via EMT is a crucial factor in PVR pathogenesis [14]. During EMT, RPE cells transition from their regular cuboidal morphology to the characteristic spindle shape of myofibroblasts. This change involves the initial loss of tight junctions, particularly zonula occludens (ZO)-1 [14]. The role of α-smooth muscle actin (α-SMA, encoded by *ACTA2*) and fibronectin (FN, encoded by *FN1*) as critical markers of myofibroblasts is well-established [15, 16]. An analysis of human PVR membranes has shown that most α-SMA-expressing myofibroblasts originate from cytokeratin-positive RPE cells [17]. Furthermore, various studies have reported the presence of FN in preretinal membranes [18], epiretinal membranes, and vitreous in patients with PVR [19].

Currently, a significant challenge in the field of PVR is the development of strategies to prevent disease progression. Given the importance of RPE-EMT in PVR, numerous studies have explored its underlying mechanisms. This intricate RPE-EMT process is accompanied by RPE cell proliferation [20–23] and is regulated at multiple levels by transcription factors, growth factors, non-coding RNAs, and other factors [24–26]. However, the lack of appropriate animal models and understanding of the mechanisms controlling RPE-EMT and proliferation have contributed to the unavailability of effective therapeutic and preventive strategies for PVR. Therefore, there is an urgent need to develop appropriate animal models and to identify additional factors that can prevent RPE-EMT and the progression of PVR.

Our research group has studied the function and dysfunction of RPE cells. To identify factors and gain a deeper understanding of the genes and pathways involved in the RPE-EMT process, we isolated RPE tissue from the eyes of donors and subjected it to enzymatic dissociation and passage to induce EMT and proliferation. Next, RNA sequencing (RNA-Seq) was used to analyze differential transcriptome profiles. This approach confirmed previously reported alterations in EMT-related genes and uncovered novel genes that had not been previously implicated. Because the EMT of RPE in pathological conditions shares commonalities with malignancy-associated EMT, including increased motility and proliferation, we investigated whether cancer-related genes changed in this transcriptome analysis played a role in RPE-EMT. One gene of particular interest is absent in melanoma 2 (*AIM2*), which is significantly downregulated in cultured primary human RPE (phRPE) cells compared to native RPE cells.

AIM2 is a member of the pyrin and HIN domain-containing (PYHIN) proteins [27] and was initially associated with the regulation of tumorigenicity in a human melanoma model [28]. In the immune system, AIM2 functions as a sensor for aberrant cytosolic double-stranded DNA (dsDNA) and stress conditions, playing a crucial role in the defense against bacterial and viral pathogens, as well as in autoimmunity caused by aberrant self-DNA [29–33]. Upon binding with aberrant cytoplasmic dsDNA, AIM2 recruits the adaptor protein, apoptosis speck-like protein (ASC), leading to the activation of caspase-1 and subsequent cleavage of pro-IL-1β and pro-IL-18 into their mature forms. This results in gasdermin-D-mediated programmed cell death, known as pyroptosis [30, 34–36]. In contrast, AIM2 also plays a critical role in oncogenesis in an inflammasome-dependent or inflammasome-independent manner. Mutations or loss of *AIM2* have been observed in colorectal cancer [37, 38]. AIM2 is known to suppress colorectal tumorigenesis by limiting colorectal cell proliferation and EMT processes in an inflammasome-independent manner [39–42]. Additionally, AIM2 inhibits EMT or proliferation in other cancer types, such as hepatocellular carcinoma and breast cancer [43, 44]. However, whether AIM2 plays a role in RPE-EMT remains unknown.

In the present study, we explored the role of AIM2 in RPE-EMT using two distinct models. The first model involved enzymatic dissociation of phRPE monolayer into single cells, followed by culturing to induce EMT in vitro. The second model established an experimental RD model to generate a PVR-like phenotype, and the RPE-EMT during this pathological process was examined in vivo [24]. By overexpressing AIM2 in phRPE cell cultures and generating RD-induced PVR in *Aim2*-deficient mice (*Aim2*^*−/−*^) [32], we demonstrated that AIM2 inhibited RPE-EMT and PVR progression in an inflammasome-independent manner.

## Materials and methods

### Ethical approval and mice

The research involving clinical samples was approved by the ethics committee of the Eye Hospital of Wenzhou Medical University (2021-210-K-192) and following the Declaration of Helsinki. All subjects provided their written approval and consent.

Experimental mice were maintained in Wenzhou Medical University’s SPF facility. C57BL/6 J mice were purchased from Charles River Laboratories of China, and *Aim2-/-* mice were purchased from Jackson Laboratory (Stock Number: 013144). The animal care and experimental procedures were performed in compliance with the ARVO statements on the Use of Animals in Ophthalmic and Vision Research, and this study was approved by the Experimental Animal Ethics Committee of Wenzhou Medical University (wydw2021-0062).

### Cell culture, siRNAs transfection and virus infection

Primary human RPE (phRPE) cells were isolated from the donor eyes (obtained from the Eye Bank of Wenzhou Medical University & Wenzhou Eyebank). PhRPE cells were cultured in DMEM/F12 supplemented with 10% fetal bovine serum (FBS) (Gibco, Life Technologies) at 37 °C in a humidified incubator containing 5% CO2.

Two independent reported siRNAs against AIM2 (si-AIM2-1 [45] and si-AIM2-2 [46]) or negative controls (si-C) were produced by GenePharma (Shanghai, China). Their sequences are shown in Supplementary Data 1. According to the manufacturer’s protocol, RPE cells were transfected with si-C or the indicated siRNAs using LipoJet Reagent (SignaGen Laboratories).

Lentivirus overexpressing AIM2 (Lv-AIM2) was purchased from Genechem Co., Ltd (Shanghai, China), which constructed human AIM2 (NM_004833) into a Ubi-MCS-SV40-EGFP-IRES-puromycin lentiviral vector. RPE cells were infected with 1×10^8^ TU/ml Lv-AIM2 or control Lentivirus (Lv-GFP) immediately after isolation from the donor eyes. After one week of cultures, puromycin was used to screen for Lv-GFP- or Lv-AIM2-infected RPE cells.

### RNA-Seq analysis and RT-qPCR analysis

For RNA-Seq analysis, total RNAs of native RPE cells and P3 cultured phRPE cells (three biological replicates for each group) were extracted using the RNAprep Pure Micro Kit (TIANGEN Biotech Co. Ltd., Beijing, China) according to the manufacturer’s instructions. Purified RNA samples generated RNA libraries for Illumina Novaseq 6000 (PE150 sequence). Beijing Novogene (China) performed experiments and data normalization. The threshold values we used to define upregulation or downregulation were |log2 (fold change) | > 1 and adjusted *p*-value < 0.05. The Excel file containing information on differentially expressed genes, along with their corresponding fold changes and *p*-values was provided as Supplementary Data 2. Raw RNA-seq data were deposited to the Gene Expression Omnibus database (GSE282859) and are available online.

For RT-qPCR Analysis, RNA was reverse transcribed into cDNA using random primer and M-MLV reverse transcriptase (Promega Corporation, Madison, WI, USA). Real-time qPCR was performed in triplicate with Power SYBR Green PCR Master Mix on a 7500 Real-Time PCR Detection System (Applied Biosystems, Foster City, CA, USA). We normalized relative mRNA expression levels to GAPDH and analyzed them using the 2^-ΔΔCt^ method. Primers used in quantitative PCR were listed in Supplementary Data 1.

### Transwell migration assay

Pore size culture inserts (8 μm) (Transwell; Costar, Corning, CA, USA) were placed into the wells of 24-well culture plates. 6 × 10^4^ Lv-AIM2 or Lv-GFP phRPE cells were seeded in the upper chamber in 200 μl DMEM/F12. The lower chamber was filled with 600 μl DMEM/F12 supplemented with 10% FBS. After incubation at 37 °C for 18 h, the cells that had migrated through the pores were fixed with 4% PFA for 20 min and stained with crystal violet for 20 min. We then determined the number of migrated cells by counting five random fields under the microscope (Zeiss).

### Gel contraction assay

The 48-well culture plates were coated with 1% BSA solution for 2 h at 37 °C. Collagen I (rat tail; Gibco, Life Technologies) was diluted in DMEM / F12 at 1 mg/ml and incubated at 37 °C to form gel. 1.2 × 10^5^ /mL phRPE cells were resuspended in serum-free DMEM/F12 culture medium and added to the collagen gel. The collagen gel was then freed from the sides of the wells using a micropipette. The images of the gels, a visually captivating part of the process, were captured after 24 hours. The area of the gels was measured using ImageJ software.

### Experimental PVR models and MK2206 treatment

The 0.25% hyaluronate was injected into the subretinal space to induce a long-lasting rhegmatogenous retinal detachment (RD) to trigger PVR-like phenotypes in 8-week-old WT or *Aim2*^*-/-*^ mice [24]. Eyeballs were collected after 10 d for further examinations as described in the Results.

*Aim2*^*-/-*^ mice were treated topically with eye drops containing MK2206 (Targetmol, T1952) at 125 μM in artificial tears or artificial tears containing DMSO as control twice daily for 10 d after establishing the retinal detachment model. Eyeballs were collected after 10 d for further examinations as described in the Results.

### Immunofluorescence (IF) and quantification of fluorescent intensity

The IF and fluorescent intensity quantification were performed as described previously [24, 47]. Briefly, eyes were fixed in 4% paraformaldehyde (PFA) for 2 h. Cryostat sections (10 μm) were blocked with 5% bovine serum albumin at RT for 1 h. The samples were incubated overnight at 4 °C with anti-ZO-1 antibody (1: 200; Invitrogen, 402200), anti-FN antibody (1: 200; Sigma-Aldrich Corp., F7387), anti-α-SMA antibody (1: 200; Sigma-Aldrich Corp., A2547), anti-VIM antibody (1: 200; Cell Signaling Technology, 5741), anti-N-CAD antibody (1: 200; Cell Signaling Technology, 13116), anti-OTX2 antibody (1: 200; R&D Systems, AF1979), or anti-p-AKT antibody (1: 100; Cell Signaling Technology, 4060 s). The staining was revealed by appropriate secondary antibodies (Life Technologies). Immunostaining results were observed and photographed on a Zeiss confocal microscope (Zeiss, Oberkochen, Germany). The relative fluorescence intensity of FN in RPE was quantified using Image J software.

### Western blotting analysis

Proteins were extracted from the phRPE or mouse RPE using a protein lysis solution supplemented with proteinase and phosphatase inhibitors (Beyotime Institute of Biotechnology). Equivalent protein extracts were loaded and separated on 6% to 15% SDS-PAGE gels and then transferred to nitrocellulose membranes (Whatman PLC, Maidstone, UK). The membranes were probed with primary antibodies as follows: anti-AIM2 antibody (1: 1000; Abcam, 93015), anti-ZO-1 antibody (1: 1000; Invitrogen, 402200), anti-FN antibody (1: 1000; Abcam, 45688), anti-α-SMA antibody (1: 1000; Cell Signaling Technology, 19245 s), anti-VIM antibody (1: 1000; Cell Signaling Technology, 5741), anti-AKT antibody (1: 1000; Cell Signaling Technology, 4691), anti-p-AKT antibody (1: 1000; Cell Signaling Technology, 4060 s), anti-DNA-PK antibody (1: 1000; Sigma-Aldrich Corp., SAB4502385), anti-DNA-PKcs (phospho S2056) (1: 1000; Abcam, ab18192), or anti-GAPDH antibody (1: 5000; Kangchen Biotech, KC-5G4). After incubating at 4 °C overnight, most of the primary antibodies were revealed with the appropriate fluorescein-conjugated secondary antibodies (LI-COR Biosciences, Lincoln, NE, USA) at room temperature for 2 h in the dark. The protein bands were scanned using the Odyssey CLx system (LI-COR Biosciences). The detection of VIM, DNA-PK, anti-DNA-PKcs (phospho S2056) was achieved using anti-rabbit IgG, horseradish peroxidase (HRP)-linked Antibody (1: 3000; Cell Signaling Technology, 7074) at room temperature for 2 h. Proteins were visualized by Omni-ECL substrate (EpiZyme, SQ203) on a FluorChem E System (Protein Simple). Quantitative densitometry of the bands was performed using ImageJ software. All original Western blotting results are shown in Supplementary Material File.

### ELISA

ELISA was performed as described previously [29]. Briefly, IL-1β and IL-18 inflammatory factors in RPE cells were detected using a mouse IL-1β and IL-18 ELISA kit (Multi Sciences Biotech Co. Ltd., Hangzhou, China). Absorbance was read at 450 nm with a reference wavelength of 570 nm by a full-wavelength microplate reader (SpectraMax190).

### Statistical analysis

All experiments are representative of at least three independent experiments, and data are presented as mean ± SD or as median with interquartile range. Data were analyzed using GraphPad Prism version 9.0 (GraphPad Software, San Diego, CA, USA). The two-tailed Student’s *t*-test was used to make comparisons between two groups. One-way ANOVA was used to make comparisons among three groups, followed by the Bonferroni post-hoc test. Areas of epiretinal or subretinal membranes in the WT and *Aim2*^*−/−*^ mice were analyzed with the Mann–Whitney U test. *P* < 0.05 was considered statistically significant.

## Results

### Downregulation of AIM2 expression during the EMT process of human RPE cells

**Fig. 1:**
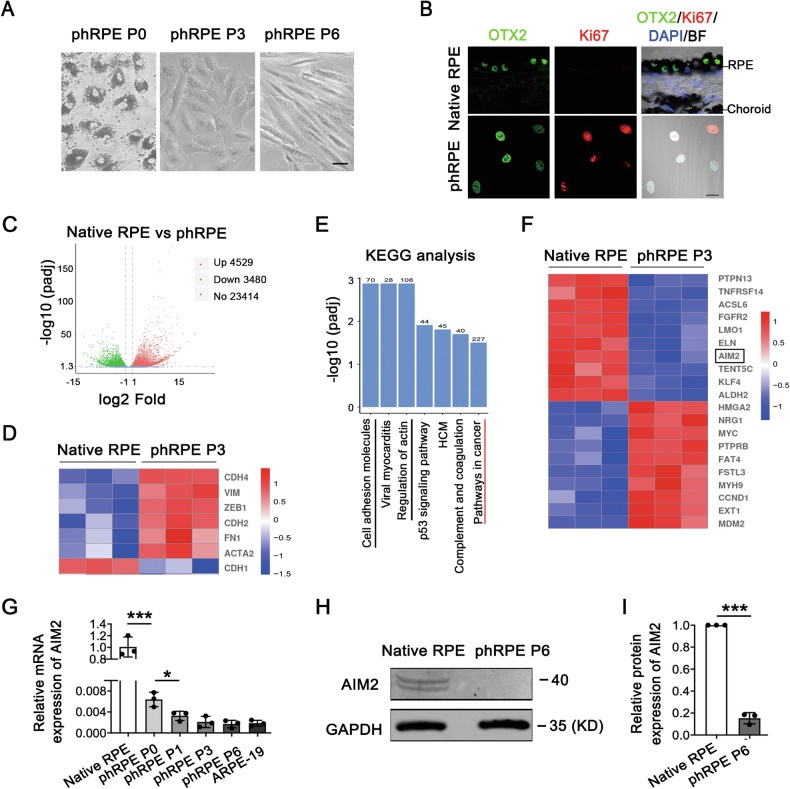
AIM2 is highly expressed in quiescent mature RPE cells and is downregulated when they initiate proliferation and undergo EMT. **A** Morphological images of phRPE cells isolated from the eyes of the donor and cultured on day one at passage 0 (P0), passage 3 (P3), and passage 6 (P6) using a phase contrast microscope. Scale bar: 20 μm. **B** Representative IF images of OTX2-labeled RPE cells and Ki67-labeled proliferative cells in native and P6 phRPE cells. Scale bar: 20 μm. **C** Volcano plot representation of DEGs detected by RNA-Seq analysis in native RPE cells from donor eyes versus P3 phRPE cells (*n* = 3). Red and green dots indicate significantly upregulated and downregulated genes, respectively. **D** Heatmap with clustering of EMT-related DEGs. **E** The bar graph of the KEGG analysis shows the top differentially regulated pathways. The black line indicates pathways related to cell adhesion- or cytoskeleton-related genes. The red line underlines cancer-related pathways. **F** Heatmap with clustering of cancer-related DEGs. **G** Relative folds of AIM2 transcript levels in native and P0, P1, P3, and P6 RPE cells, and ARPE-19 cells measured using real-time PCR analysis. *N* = 3. Data are presented as mean ± SD. * *P* < 0.05 or ***P < 0.001 by one-way ANOVA and post hoc Bonferroni’s test. **H** Representative image of AIM2 protein expression levels in native and P6 RPE cells assessed using immunoblot analysis. **I** Analysis of the band intensities in immunoblot results and normalized to GAPDH based on the results (**H**) (*n* = 3). Data are presented as mean ± SD. ***P < 0.001 by two-tailed Student’s *t*-test.

To experimentally manipulate the RPE-EMT process in human primary RPE cells, we first established an in vitro culture system using phRPE cells harvested from donor eyes. Previous studies have shown that RPE cells undergo EMT (RPE-EMT) and proliferation in primary RPE cell cultures from C57BL/6 mice [22]. Therefore, we selected a phRPE cell culture model to identify the critical factors involved in the RPE-EMT process. Initially, dissociated phRPE cells exhibited an epithelial morphology on day 1 at passage 0 (P0) (Fig. 1A, left panels). After three passages (P3), RPE cells showed an elongated morphology (Fig. 1A, middle panels), and by passage 6 (P6), the cells appeared fibroblastic (Fig. 1A, right panel). Orthodenticle homeobox 2 (OTX2) and Ki67 double immunostaining confirmed the proliferation of these P6 RPE cells (Fig. 1B).

To identify the critical factors that regulate the RPE-EMT process, we applied RNA-Seq analysis to native RPE cells from donor eyes and P3 cultured phRPE cells. Transcriptome analysis revealed an enrichment of 4529 genes and a reduction in 3480 genes, as illustrated in the volcano plot (Fig. 1C). The heatmap showed significant changes in EMT-related genes, such as *CDH4, VIM, ZEB1, CDH2, FN1, ACTA2*, and *CDH1* (Fig. 1D). These observations indicated that RPE-EMT occurred during culture. Kyoto Encyclopedia of Genes and Genome (KEGG) analysis based on RNA-Seq showed that the top molecular pathways, including cell adhesion and actin cytoskeleton, were differentially regulated during this process. Cancer pathways were also among the top molecular pathways identified (Fig. 1E). Among the heatmap representations of cancer-associated genes, *AIM2* was significantly downregulated in P3 phRPE cells (Fig. 1F). We further analyzed changes in *AIM2* expression among native RPE cells, passaged phRPE cells, and ARPE-19 cells using RT-PCR. As shown in Fig. 1G, native RPE cells exhibited high levels of *AIM2* expression, which decreased dramatically after culture. The expression levels in P3 and P6 RPE cells were comparable to those in ARPE-19 cells (Fig. 1G). Immunoblot analysis also confirmed high AIM2 expression in native RPE cells, which was almost absent in cultured RPE cells at P6. (Fig. 1H and I). These results suggest that *AIM2* expression is downregulated during proliferation and EMT of RPE cells.

### AIM2 suppresses RPE-EMT

As mentioned above, the significant decrease in *AIM2* expression during the initial stages of proliferation and RPE-EMT prompted us to explore whether AIM2 plays a functional role in RPE-EMT. First, we transfected phRPE cells with two independent siRNAs against AIM2 (si-AIM2-1 and si-AIM2-2) or with negative controls (si-C) to determine whether the decrease in AIM2 promoted the proliferation and EMT of RPE cells. The transfection was performed 2 d after RPE isolation from donor eyes. We evaluated EMT-associated genes 5 d after transfection (P0, passage 0) or 10 d after transfection (P1, passage 1). The disassociated RPE cells in primary culture showed rapid changes in EMT-associated genes. The expression levels of *CDH1*, *CDH2*, and *VIM* in P0 phRPE were similar to those in P3 phRPE, whereas the expressions of *FN1* and *ACTA2* in P0 phRPE were higher than that in native phRPE, and lower than that in P3 phRPE. Transfection with the AIM2 siRNAs led to a reduction in *AIM2* expression levels and an increase in *FN1* and *ACTA2* levels in P0 phRPE (Supplementary Fig. S1A). Additionally, the decrease in *AIM2* expression resulted in an enhanced proliferation rate, as confirmed by Ki67 staining (Supplementary Fig. S1B and C).

**Fig. 2:**
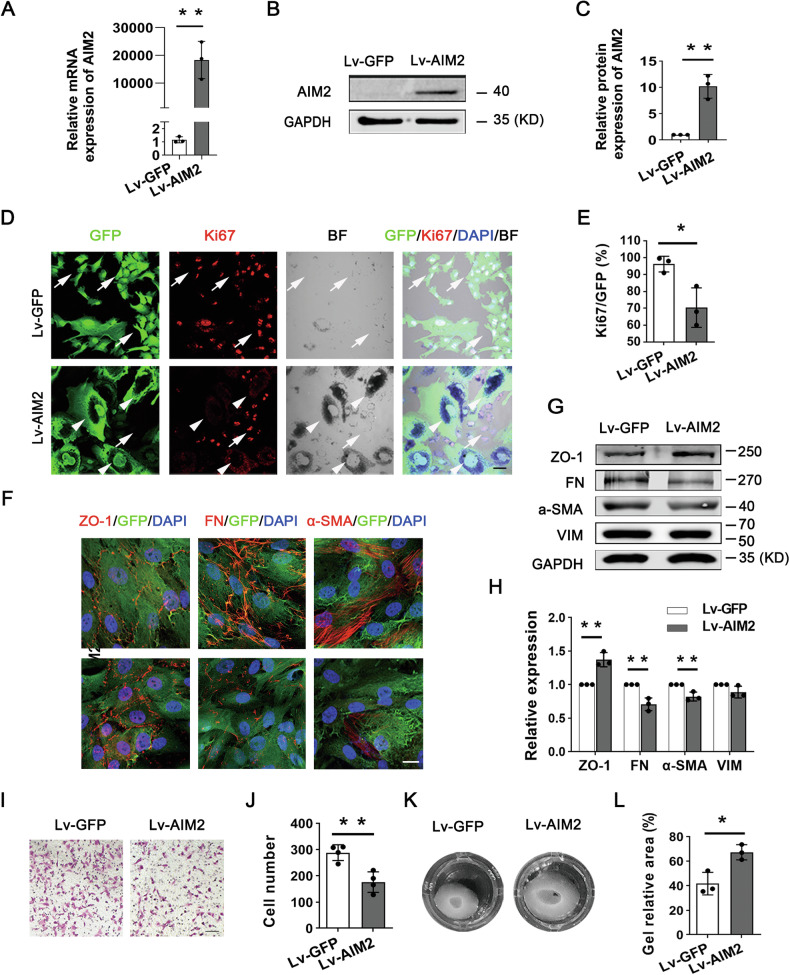
Overexpression of AIM2 inhibits phRPE cell proliferation and EMT in cell cultures. **A** Real-time PCR analysis of AIM2 mRNA levels in phRPE cells infected with a negative control lentivirus (Lv-GFP) or a lentivirus overexpressing AIM2 (Lv-AIM2). *N* = 3. Data are presented as mean ± SD. **P < 0.01 by two-tailed Student’s *t*-test. **B** Representative immunoblot analysis of AIM2 protein levels in Lv-GFP and Lv-AIM2 phRPE cells. **C** Quantification of the band intensities in immunoblot results and normalization to GAPDH based on the results (**B**) (*n* = 3). Data are presented as mean ± SD. **P < 0.01 by two-tailed Student’s *t*-test. **D** Double immunostaining of GFP and Ki67 in phRPE cells infected with Lv-GFP or Lv-AIM2. Overexpressing cells of Lv-GFP or Lv-AIM2 were marked by co-expression of GFP. White arrows indicate low-pigmented and Ki67-positive phRPE cells in Lv-GFP cells and uninfected cells in the Lv-AIM2 cells. White arrowheads indicate high-pigmented, Ki67-negative, and AIM2-overexpressing phRPE cells in Lv-AIM2 cells. Scale bar: 20 μm. **E** Quantification of Ki67-/GFP-positive cells based on the results (**D**) (*n* = 3). Data are presented as mean ± SD. *P < 0.05 by two-tailed Student’s *t*-test. **F** Representative confocal IF images of EMT-related proteins in Lv-GFP and Lv-AIM2 phRPE cells. Scale bar: 20 μm. **G** Representative immunoblot analysis of EMT-related proteins in Lv-GFP and Lv-AIM2 phRPE cells. **H** Quantification of the band intensities in immunoblot results and normalization to GAPDH (*n* = 3). Data are presented as mean ± SD. **P < 0.01 by two-tailed Student’s *t*-test. Representative image (**I**) and quantification (**J**) of Transwell migration assay of Lv-GFP and Lv-AIM2 phRPE cells. Scale bar: 50 μm. *N* = 4. Data are presented as mean ± SD. **P < 0.01 by two-tailed Student’s t-test. Representative image (**K**) and quantification (**L**) of collagen gel contraction assay of Lv-GFP and Lv-AIM2 phRPE cells. *N* = 3. Data are presented as mean ± SD. *P < 0.05 by two-tailed Student’s *t*-test.

Furthermore, to determine whether the overexpression of AIM2 can prevent the proliferation and EMT of RPE cells, we infected phRPE with Lentivirus-AIM2 (Lv-AIM2) immediately after isolating them from the eyes of donor. After puromycin selection of Lv-GFP- or Lv-AIM2-infected phRPE cells (Lv-GFP-RPEs or Lv-AIM2-RPEs, respectively), the Lv-AIM2-RPEs showed high levels of *AIM2* expression (Fig. 2A, B, and C). Double immunostaining for GFP and Ki67 was used to compare cell proliferation between uninfected RPE cells and Lv-GFP- or Lv-AIM2-RPEs. Both uninfected and Lv-GFP-RPEs exhibited a proliferative status. In contrast, Lv-AIM2-RPEs showed low proliferation (Fig. 2D and E). Next, we analyzed EMT markers in Lv-GFP-RPEs and Lv-AIM2-RPEs using immunoblotting and immunostaining. As shown in Fig. 2F–H, ZO-1 was upregulated in the Lv-AIM2 group compared with that in the Lv-GFP group, and proteins associated with the mesenchymal cell state, such as FN and α-SMA, were significantly inhibited in the Lv-AIM2 group. VIM showed a slight decrease in the Lv-AIM2 group compared with that in the Lv-GFP group. Transwell assays were performed to evaluate the migratory ability of Lv-GFP-RPEs and Lv-AIM2-RPEs. As shown in Fig. 2I and J, overexpression of AIM2 significantly inhibited the migration of phRPE cells. In the collagen gel contraction assay, AIM2 overexpression hampered collagen gel contraction mediated by phRPE cells (Fig. 2K and L). Therefore, these results indicate that the overexpression of AIM2 represses the proliferation and EMT of RPE cells.

### AIM2 deficiency leads to morphological changes and increased FN expression in RPE cells under physiological conditions

**Fig. 3:**
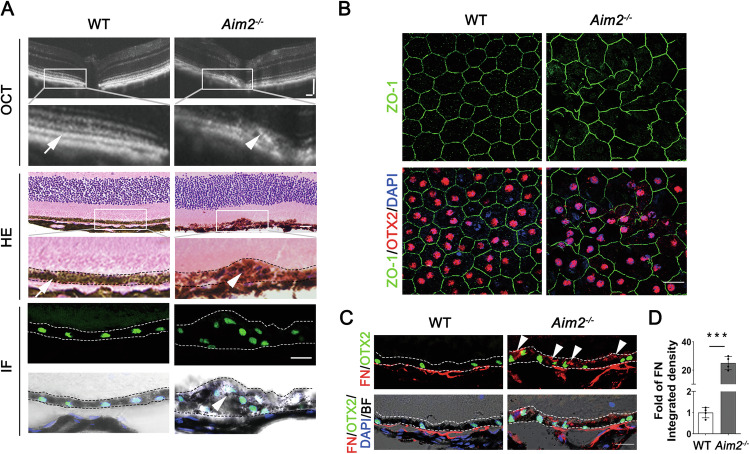
AIM2-deficient RPE cells undergo morphological changes and extracellular matrix remodeling under physiological conditions. **A** OCT scanning, HE staining, and IF assay for OTX2 labeling RPE cells in 8-week-old WT and *Aim2*^*−/−*^ RPE cells. Two dotted lines frame the RPE position. White arrows indicate the monolayer of WT RPE. White arrowheads indicate the multilayers of *Aim2*^*−/−*^ RPE. Scale bars of OCT images: 100 μm. Scale bars of HE and IF images: 20 μm. **B** Representative images of IF assay for ZO-1 (green) and OTX2 (red) in RPE flat mounts of 8-week-old WT and *Aim2*^*−/−*^ mice. Scale bar: 20 μm. **C** Representative images of the IF assay for FN (red) and OTX2 (green) in the RPE cryostat sections of 8-week-old WT and *Aim2*^*−/−*^ mice. White arrowheads indicate the increased expression of FN in *Aim2*^*−/−*^ RPE. Scale bar: 20 μm. **D** Quantifying the integrated density of FN based on the results (**C**). *N* = 3. Data are presented as mean ± SD. ***P < 0.001 by two-tailed Student’s *t*-test.

To investigate whether AIM2 regulates RPE-EMT in vivo, we evaluated the RPE status in *Aim2*^*−/−*^ mice. Although wild-type (WT) mice typically exhibited a monolayer of RPE cells, *Aim2*^*−/−*^ mice showed abnormally localized multilayers of RPE cells (Fig. 3A). IF analyses of ZO-1 and FN revealed morphological alterations (Fig. 3B) and an elevation in FN expression in *Aim2*^*−/−*^ RPE cells (Fig. 3C and D). In addition, *Aim2*^*−/−*^ RPE cells showed no proliferation, as indicated by the absence of Ki67 expression (Supplementary Fig. S2A and B). These results suggest that *Aim2*^*−/−*^ RPE cells undergo extracellular matrix remodeling rather than proliferation under physiological conditions.

### AIM2 deficiency promotes RPE-EMT, leading to severe experimental PVR progression

**Fig. 4:**
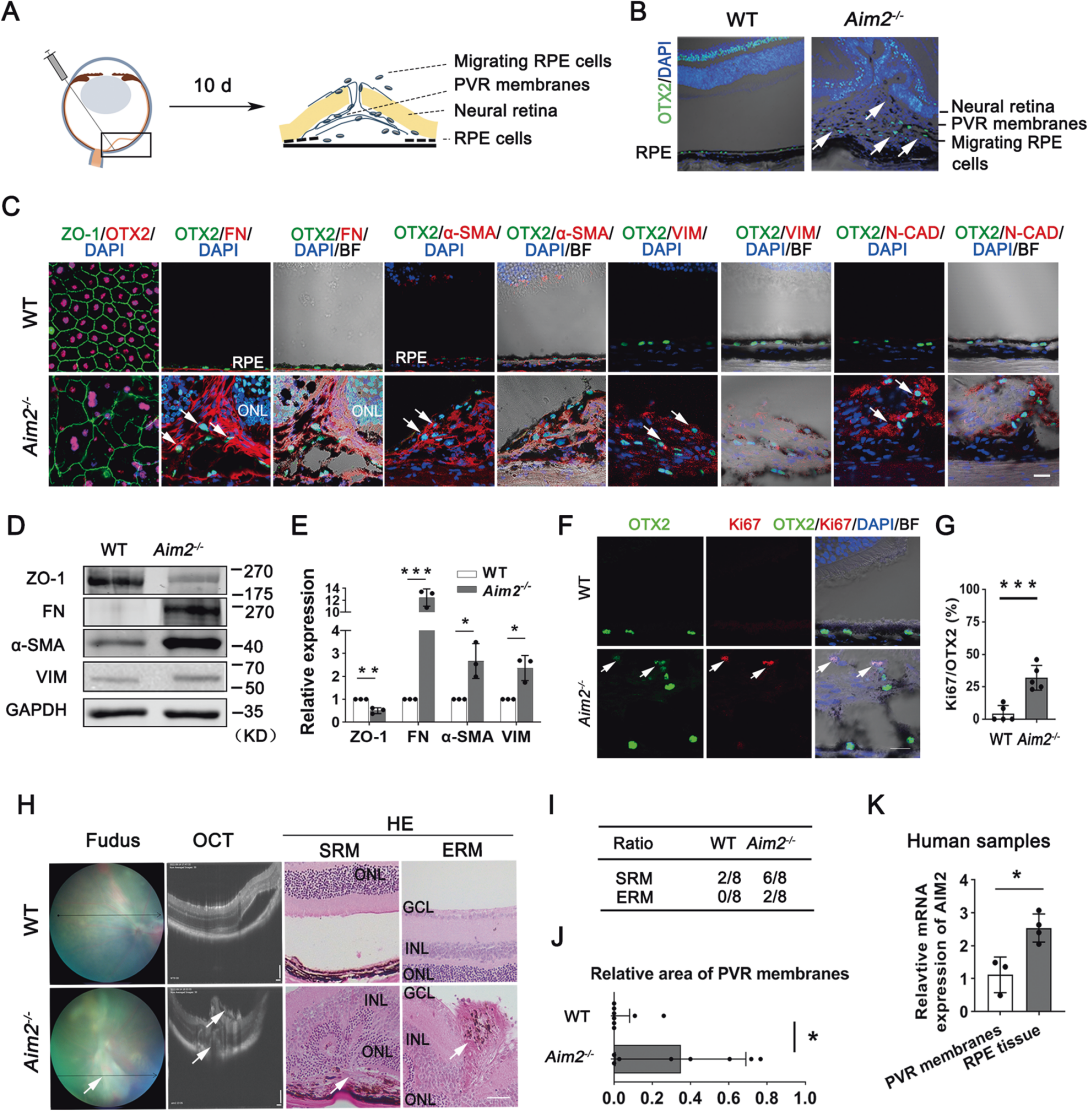
AIM2 deficiency promotes proliferation and EMT of RPE cells, leading to severe progressive experimental PVR. **A** Schematic representation of an experimental RD-induced PVR model in mice. Mice were subretinally injected with 0.25% sodium hyaluronate for 10 d to induce RD and trigger PVR progression. **B** Immunostaining for OTX2 (green) in RD-induced PVR in WT or *Aim2*^*−/−*^ mice (hereafter referred to as WT or *Aim2*^*−/−*^ PVR mice). Arrows indicate OTX2-positive RPE cells, which detached from Bruch’s membrane, migrating into *Aim2*^*−/−*^ subretinal space and forming a subretinal membrane. Scale bar: 50 μm. **C** Representative images for ZO-1 (green) and OTX2 (red) in RPE flat mounts (left panel), and representative images for FN, α-SMA, VIM, and N-CAD (middle and right panels) in cryostat sections of WT and *Aim2*^*−/−*^ PVR mice. Arrows indicate OTX2-positive RPE cells migrating into the subretinal space, which express mesenchymal cell markers, FN, α-SMA, VIM, and N-CAD. Scale bar: 20 μm. ONL, photoreceptor outer nuclear layer. **D** Representative immunoblot analysis of EMT-related proteins in RPE cells in WT and *Aim2*^*−/−*^ PVR mice. **E** Quantification of the band intensities in immunoblot results and normalized to GAPDH (*n* = 3). Data are presented as mean ± SD. *P < 0.05, **P < 0.01, and ***P < 0.001 by two-tailed Student’s *t* test. **F** Double immunostaining for OTX2 and Ki67 in WT and *Aim2*^*−/−*^ PVR mice. Arrows indicate Ki67-positive RPE cells in *Aim2*^*−/−*^ PVR mice. Scale bar: 20 μm. **G** Quantifying the Ki67-positive RPE cells based on the results (**F**) (*n* = 5). Data are presented as mean ± SD. ***P < 0.001 by two-tailed Student’s *t*-test. **H** Fundus photographs (left panels), OCT scanning images (middle panels), and HE stains (right panels) in WT and *Aim2*^*−/−*^ PVR mice. Arrows indicate epiretinal or subretinal membranes in the *Aim2*^*−/−*^ mice. Scale bars: images of OCT (100 μm) and HE (50 μm). INL inner nuclear layer, ONL photoreceptor outer nuclear layer, GCL ganglion cell layer, ERM epiretinal membrane, SRM subretinal membrane. **I** Occurrence rate of epiretinal or subretinal membranes in WT and *Aim2*^*−/−*^ PVR mice. **J** Area analysis of epiretinal or subretinal membranes in the WT and *Aim2*^*−/−*^ mice. Data are presented as median with interquartile range. *P < 0.05 by Mann–Whitney U test. **I**, **J** *n* = 8 mice, respectively. **K** Relative changes of *Aim2* transcripts in native RPE cells from donor eyes (*n* = 4) and PVR membranes from surgery (*n* = 3). Data are presented as mean ± SD. *P < 0.05 by two*-*tailed Student’s *t*-test.

As RPE-EMT is vital in the pathological progression of PVR, we investigated the effects of *Aim2* deficiency on RPE-EMT in a retinal detachment (RD)-induced PVR mouse model. To mimic human PVR pathology, rhegmatogenous RD was induced by surgically injecting 0.25% sodium hyaluronate into the subretinal space to trigger PVR-like phenotypes in mice (Fig. 4A). After 10 d, WT mice showed RD with an intact RPE layer, whereas *Aim2*^*−/−*^ RPE cells migrated to the subretinal space, leading to the formation of PVR membranes and localized retinal traction and distortion (Fig. 4B). As shown in Fig. 4C, immunostaining analyses of ZO-1 showed that RPE cells displayed irregular and distorted morphology in the RD-induced PVR model in *Aim2*^*−/−*^ mice (*Aim2*^*−/−*^ PVR mice), whereas FN, α-SMA, VIM, and N-CAD were highly expressed in *Aim2*^*−/−*^ RPE cells that migrated into the subretinal space (Fig. 4C). Immunoblot data further confirmed decreased levels of ZO-1 and increased levels of FN, α-SMA and VIM in the *Aim2*^*−/−*^ RPE cells (Fig. 4D and E). In addition, some *Aim2*^*−/−*^ RPE cells that migrated in the subretinal space exhibited proliferation (Fig. 4F and G).

To further evaluate the contribution of changes in RPE status to the development of PVR in *Aim2*^*−/−*^ mice, the occurrence of PVR was visualized using color fundus and optical coherence tomography images and confirmed by Hematoxylin-Eosin staining. As shown in Fig. 4H, epiretinal and subretinal membrane formation was observed, resulting in retinal contraction and folding in *Aim2*^*−/−*^ mice. To evaluate the severity and rate of PVR, the occurrence and area of PVR membranes were quantified. In *Aim2*^*−/−*^ PVR mice, the majority developed subretinal membranes (SRMs), and a smaller proportion developed epiretinal membranes (ERMs), whereas only a minimal number of WT PVR mice developed SRMs, and none developed ERMs (Fig. 4I). Moreover, *Aim2*^*−/−*^ PVR mice showed more significant areas of epiretinal and subretinal membranes (Fig. 4J). We also examined *AIM2* expression in PVR membranes from clinical PVR samples and native RPE cells from donor eyes. As shown in Fig. 4K, *AIM2* expression was lower in PVR membranes than that in native RPE cells. These results indicate that *AIM2* deficiency promotes EMT and proliferation of RPE cells, leading to severe PVR progression.

### AIM2 deficiency leads to AKT activation in an inflammasome-independent manner

**Fig. 5:**
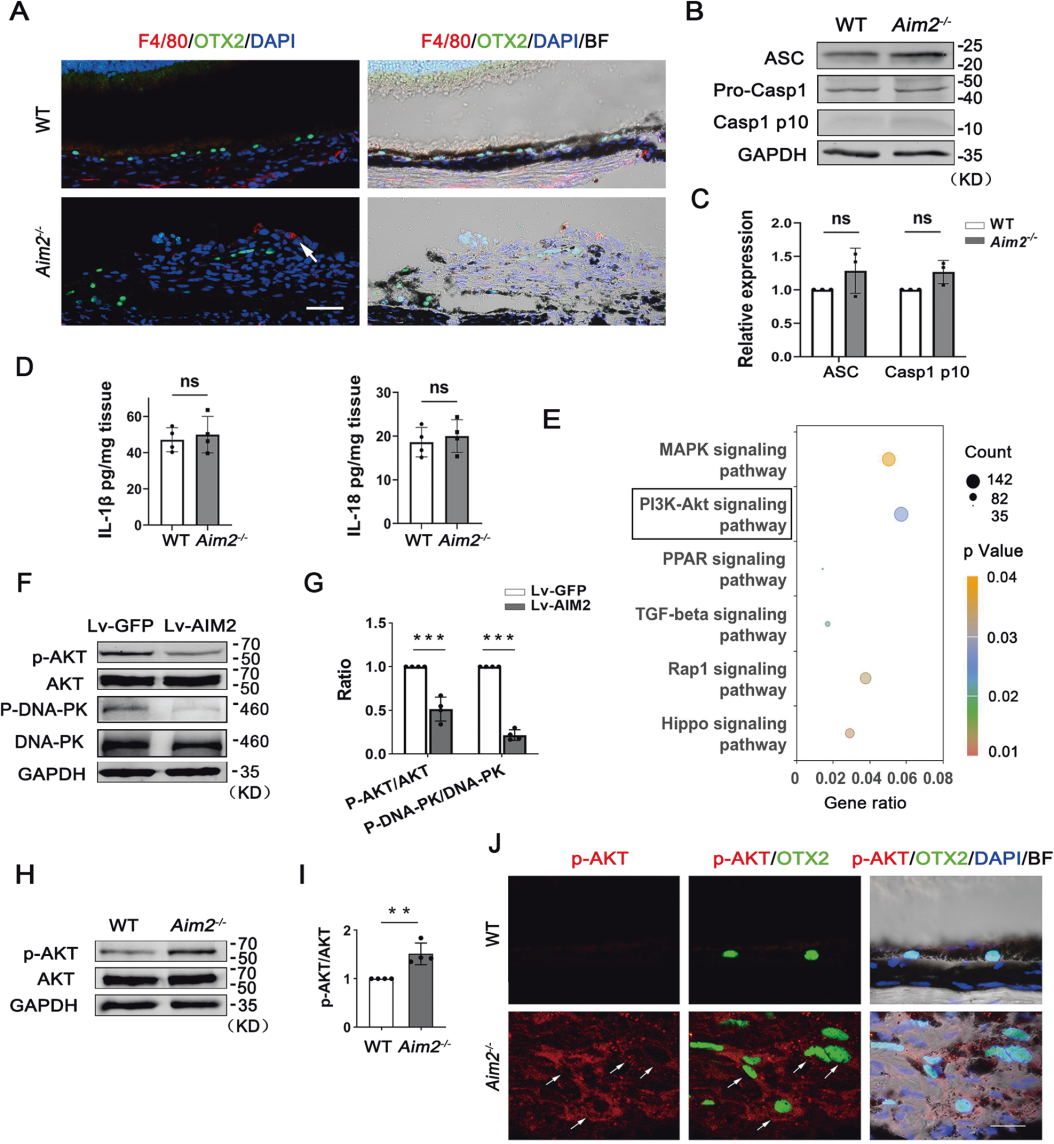
AIM2 deficiency leads to AKT activation in an inflammasome-independent manner in RPE cells in *Aim2*^*−/−*^ PVR mice. **A** Immunostaining for OTX2 (green) and F4/80 (red) in WT or *Aim2*^*−/−*^ PVR mice. The arrow points to the F4/80-positive macrophage cell. Scale bar: 50 μm. Immunoblot analysis (**B**) and quantification of band intensity (**C**) of ASC, Casp1 p10, and GAPDH (as protein-loading control) in PVR membranes of WT and *Aim2*^*−/−*^ PVR mice (*n* = 3). Data are presented as mean ± SD. Ns: not significant by two-tailed Student’s *t*-test. **D** Analyses of IL-1β and IL-18 using ELISA in PVR membranes of the WT and *Aim2*^*−/−*^ mice (*n* = 4). Data are presented as mean ± SD. Ns: not significant by two-tailed Student’s *t*-test. **E** Dot graph of KEGG analysis of RNA-Seq in native phRPE cells versus P3 phRPE cells shows the differentially regulated signaling pathways. The gene ratio in the x-axis indicates the ratio of the number of DEGs annotated on the KEGG pathway to the total number of differential genes. Counts indicate the numbers of DEGs annotated by KEGG pathway analysis. The ratio of altered genes in the PI3K-Akt signaling pathway is 0.057 (142/2482 genes). Immunoblot analysis (**F**) and quantification of band intensity (**G**) of AKT, p-AKT, DNA-PK, P-DNA-PK, and GAPDH in Lv-GFP and Lv-AIM2 phRPE cells. (*N* = 4). Data are presented as mean ± SD. ***P < 0.001 by two-tailed Student’s t-test. Immunoblot analysis (**H**) and quantification of band intensity (**I**) of AKT, p-AKT, and GAPDH in RPE cells of WT or *Aim2*^*−/−*^ PVR mice (*n* = 3). Data are presented as mean ± SD. **P < 0.01 by two-tailed Student’s *t*-test. **J** Double immunostaining for OTX2 labeling RPE cells and p-AKT in WT and *Aim2*^*−/−*^ PVR mice. White arrows indicate that the OTX2-positive cells have the increased levels of p-AKT in *Aim2*^*−/−*^ PVR mice. Scale bar: 20 μm.

Following rhegmatogenous RD, macrophages can be found in the subretinal and epiretinal PVR [48]. To determine whether AIM2 regulates EMT, proliferation of RPE, and PVR pathogenesis through macrophages, we used the macrophage marker F4/80 to label macrophages in subretinal PVR. IF analysis of F4/80 revealed the presence of a small number of macrophages in the subretinal membranes (Fig. 5A). Immunoblot analysis showed no significant changes in caspase-1 proteolytic cleavage (P10) or the levels of IL-1β and IL-18 in the PVR membranes, which consisted of RPE cells and a few macrophages in *Aim2*^*−/−*^ experimental PVR model (Fig. 5B–D), suggesting that AIM2 influences PVR progression in an inflammasome-independent manner.

To investigate which downstream pathways AIM2 regulates, we performed KEGG analysis based on RNA-Seq data from native phPRE cells versus cultured phRPE cells. Interestingly, the results showed that some altered genes were involved in different signaling pathways, such as MAPK (Gene Ratio = 125/2482, *P* = 0.039), PI3K-AKT (Gene Ratio = 142/2482, *P* = 0.024), and TGF-beta (Gene Ratio = 42/2482, *P* = 0.021) signaling pathways (Fig. 5E). Notably, 142 genes out of 2482 changed genes identified by KEGG analysis belonged to the PI3K-AKT signaling pathway (Fig. 5E and Supplementary Fig. S3). Because of the recognized importance of the PI3K/AKT pathway in regulating cell proliferation, migration, and the expression of EMT-related markers [49–51], we evaluated AKT phosphorylation in *AIM2*-overexpressing phRPE and RPE cells from *Aim2*^*−/−*^ mice. Compared with controls, *AIM2*-overexpressing phRPE cells showed decreased phosphorylation of AKT at Ser473 and a reduction in phosphorylation of DNA-dependent protein kinase (DNA-PK), which regulates AKT phosphorylation (Fig. 5F and G). In addition, RPE cells in *Aim2*^*−/−*^ PVR mice showed increased phosphorylation of AKT compared with that in their WT counterparts, as confirmed by immunoblot analysis (Fig. 5H and I). In addition, the level of p-AKT was increased in OTX2-positive RPE cells in *Aim2*^*−/−*^ PVR mice (Fig. 5J). These results indicate that *AIM2* deficiency leads to AKT activation in RPE cells during experimental PVR in an inflammasome-independent manner, and that overexpression of *AIM2* in RPE cells can repress AKT activation.

### Inhibition of AKT activation suppresses RPE-EMT and experimental PVR progression

**Fig. 6:**
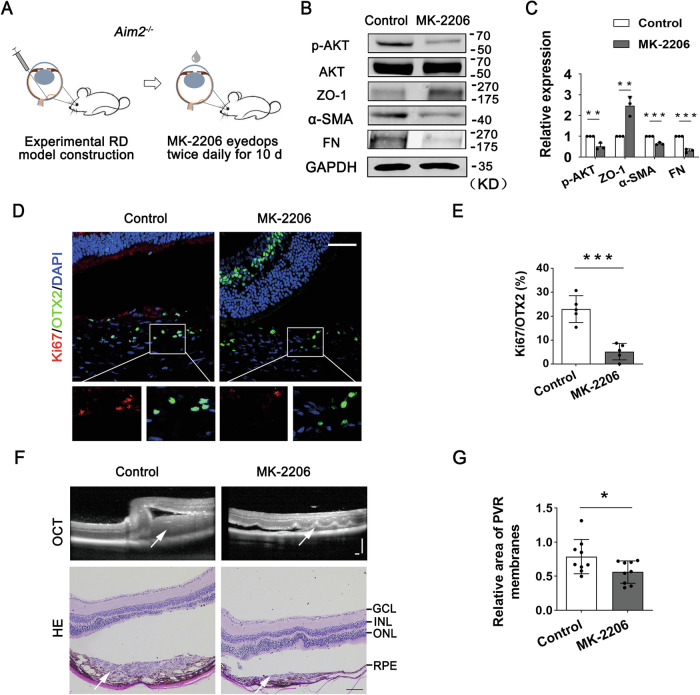
AKT inhibitor suppresses RPE-EMT and proliferation and prevents the subretinal membrane formation in *Aim2*^*−/−*^ PVR mice. **A** Schematic representation of AKT-specific inhibitor MK2206 treatment in *Aim2*^*−/−*^ PVR mice. Eye drops of control (artificial tears containing DMSO) or MK2206 were applied topically twice daily for 10 d. Eyes were harvested and analyzed after 10 d. Immunoblot analysis (**B**) and quantification of band intensity (**C**) of p-AKT, EMT-related proteins, and GAPDH in RPE cells from *Aim2*^*−/−*^ PVR mice with the indicated conditions (*n* = 3). Data are presented as mean ± SD. **P < 0.01, ***P < 0.001 by two-tailed Student’s *t*-test. **D** Double immunostaining for OTX2 (green) and Ki67 (red) in *Aim2*^*−/−*^ PVR mice with the indicated conditions. Scale bar: 50 μm. **E** Quantification of the Ki67-positive RPE cells based on the results (**D**). (*n* = 5). Data are presented as mean ± SD. ***P < 0.001 by two-tailed Student’s *t*-test. **F** Representative images of OCT scanning and HE stains of *Aim2*^*−/−*^ PVR mice with indicated conditions. Arrows indicate subretinal membranes. GCL ganglion cell layer, INL inner nuclear layer, ONL photoreceptor outer nuclear layer, RPE retinal pigment epithelium. The scale bars for OCT and HE staining: 200 and 100 μm, respectively. **G** Area analysis of PVR membranes in *Aim2*^*−/−*^ PVR mice administrated with control (*n* = 9 mice) or MK-2206 (*n* = 9 mice). Data are presented as mean ± SD. *P < 0.05 by two-tailed Student’s *t*-test.

To confirm whether overactivated AKT phosphorylation is required for increased RPE-EMT, proliferation, and PVR progression, we used a specific AKT inhibitor, MK2206, to suppress AKT activation in *Aim2*^*−/−*^ PVR mice. After establishing the RD-induced PVR model in both WT and *Aim2*^*−/−*^ mice, the mice were treated with eye drops containing MK2206 twice daily for 10 d (Fig. 6A). Western blotting results showed that AKT phosphorylation was partially inhibited in the RPE cells of *Aim2*^*−/−*^ PVR mice following MK2206 administration. Additionally, the epithelial marker ZO-1 expression was increased, and the mesenchymal biomarkers, FN and α-SMA, were significantly decreased following MK2206 administration (Fig. 6B and C). Immunostaining results showed that RPE cell proliferation in the *Aim2*^*−/−*^ PVR mice was inhibited by MK2206 (Fig. 6D and E). Moreover, MK2206 suppressed the area of PVR membranes in *Aim2*^*−/−*^ PVR mice (Fig. 6F and G). These results suggest that the inhibition of AKT activation suppresses the RPE-EMT and proliferation, thereby preventing experimental PVR progression.

## Discussion

RPE-EMT plays a crucial role in the development of various retinal diseases, including AMD and PVR [8, 9]. In the present study, we provided several lines of evidence that *AIM2* suppresses EMT in RPE and experimental PVR. First, we showed that *AIM2* was highly expressed in native phRPE cells but decreased significantly during the proliferation and EMT processes in cultured RPE cells. Second, the overexpression of *AIM2* in phRPE cells effectively inhibited both proliferation and EMT, whereas RPE cells in *Aim2*^*−/−*^ mice exhibited morphological changes and extracellular matrix remodeling under physiological conditions. Third, in an experimental PVR mouse model, AIM2 deficiency promoted EMT and the proliferation of RPE cells, thereby leading to severe PVR progression. Fourth, *AIM2* negatively regulates AKT phosphorylation in both AIM2-overexpressing phRPE and RPE cells in *Aim2*^*−/−*^ mice. Fifth, eye drops containing an AKT inhibitor inhibited AKT phosphorylation and attenuated RPE-EMT and PVR progression in *Aim2*^*−/−*^ PVR mice. Collectively, these findings indicate that high levels of AIM2 are crucial for maintaining RPE homeostasis to prevent the proliferation and EMT of RPE cells.

Considering these findings, establishing human primary RPE cells and an RD-induced PVR model in *Aim2*^*−/−*^ mice may provide more appropriate models for understanding the regulation of EMT and the proliferation of RPE cells. The processes of RPE-EMT and proliferation in retinal diseases are closely intertwined [20–23]. Investigating the mechanisms underlying these cellular activities in both in vitro human primary RPE models and in vivo mouse models is important for understanding the pathogenesis of related retinal diseases.

In our RD-induced PVR mouse model, *Aim2*^*−/−*^ RPE cells showed increased levels of EMT, and some RPE cells that migrated in the subretinal space exhibited proliferation, comparable to that observed in patients with PVR. Under physiological conditions, *Aim2*^*−/−*^ mice exhibited localized multilayers of RPE characterized by multinucleation and distorted ZO-1, coupled with upregulated FN, rather than proliferation. This manifestation closely resembles the features observed in patients with AMD, such as fully pigmented, multinucleated, non-uniform, and localized thickening of the RPE layers [9]. The key difference between the *Aim2*-deficient PVR model and physiological conditions is that growth factors from the vitreous body in the PVR model make AIM2-deficient RPE cells more prone to proliferation and EMT. Under physiological conditions, the RPE tightly contacts the surrounding tissues and neural retinas, thereby blocking contact between RPE cells and potential growth factors from the vitreous body, which helps keep Aim2-deficient RPE cells non-proliferative. These aspects may explain why the RPE of patients with AMD and *Aim2*^*−/−*^ mice under normal conditions exhibit ECM remodeling and migratory characteristics rather than proliferation. Although both in vitro and in vivo models can be used to study the underlying mechanisms of EMT and cell proliferation, some variations exist. In particular, in vivo models are better suited for replicating certain features of natural scenarios.

AIM2, a molecule with broad expression, has been the focus of several previous studies. These studies have shown that AIM2 inflammasomes can be activated in cultured Jak2VF macrophages [52] and in macrophages that have internalized necrotic cell DNA [53]. Moreover, AIM2 has been reported to regulate macrophage infiltration, thus leading to inflammation and tissue damage [29, 53, 54]. Research on *Aim2*^*−/−*^ mice has revealed reduced macrophage infiltration and associated histopathological changes in models of dry eye disease [29], polyarthritis-like disease [54], and unilateral ureteral obstruction [53]. Macrophages, crucial cells in the pathogenesis of fibrotic diseases [55, 56], have also been observed in the subretinal and epiretinal areas in PVR following rhegmatogenous RD [48]. Our IF analyses of F4/80 revealed a small number of macrophages in the subretinal membranes of *Aim2*^*−/−*^ PVR mice, suggesting a limited role of macrophages in this PVR model. Additionally, previous studies have shown that the activation of AIM2 inflammasomes in macrophages leads to increased IL-1β or IL-18 levels [29, 53, 57, 58]. However, no significant alterations in IL-1β and IL-18 levels were observed in the PVR membranes of *Aim2*^*−/−*^ PVR mice, suggesting that AIM2 may not influence PVR pathogenesis through macrophages.

In the present study, we showed that AIM2 suppressed RPE-EMT in an inflammasome-independent manner. AIM2 exerts both inflammasome-dependent and inflammasome-independent functions in health and disease [34, 39, 40]. Emerging evidence has indicated that activation of the AIM2 inflammasome triggers TGF-β release in an IL-1α-dependent manner in peripheral blood mononuclear cells from patients with idiopathic pulmonary fibrosis [59]. TGF-β is a well-known pro-EMT and pro-fibrotic factor in the RPE [60, 61]. We examined whether AIM2 regulated TGF-β to influence RPE-EMT and found that *AIM2* overexpression did not alter TGF-β expression levels (data not shown). Moreover, the downregulation of *AIM2* expression in the membranes of patients with PVR eliminates the possibility of AIM2 inflammasome activation during PVR progression. These results suggest that the function of AIM2 in RPE-EMT is inflammasome-independent. Previous studies reported that AIM2 attenuates AKT phosphorylation in Treg cells and colons in an inflammasome-independent manner [40, 62]. The results of this study further demonstrate that AIM2 inhibits the EMT of RPE cells via AKT activation. It has been reported that AIM2 physically interacts with and limits the activation of DNA-PK, a member of the PI3K-related family, to suppress AKT phosphorylation. Conversely, the absence of AIM2 leads to DNA-PK-mediated AKT activation [40]. Our data further supported this, showing a significant decrease in p-DNA-PK (S2056) in Lv-AIM2-RPEs compared with that observed in Lv-GFP-RPEs, suggesting that AIM2 inhibits AKT phosphorylation through DNA-PK in RPE cells.

A previous report demonstrated that the vitreous stimulates AKT activation in RPE cells derived from the epiretinal membranes of a patient with PVR [63]. KEGG analysis of RNA-Seq data from native phPRE cells versus cultured phRPE cells suggested that the PI3K-AKT signaling pathway is involved in the proliferation and EMT of RPE cells. Thus, the activation of AKT may play a crucial role in proliferation, EMT, and PVR development. Previous studies have highlighted the role of the PI3K/AKT pathway in regulating cell proliferation, migration, invasion, and the expression of EMT-related markers [49–51]. Specifically, AKT phosphorylation promotes FN production [64–66], which is hindered in Akt1-deficient galactose-fed mice [64] or suppressed by inhibiting the PI3K/AKT pathway [65–67]. In addition, blocking the PI3K/AKT pathway suppresses α-SMA synthesis [68]. Our results aligned with these findings, as *AIM2*-overexpressing phRPE cells exhibited reduced AKT phosphorylation, FN, and α-SMA expression. Conversely, RPE cells in *Aim2*^*−/−*^ PVR mice showed increased AKT phosphorylation and FN and α-SMA expression compared with that shown by their WT counterparts. Furthermore, treatment with the AKT inhibitor significantly decreased FN and α-SMA levels, suggesting that these proteins may be downstream targets of AKT activation.

Although the carcinoma and RPE-EMT programs are influenced by distinct signals from their respective microenvironments, they share some common characteristics. Deficiencies in AIM2 result in pathological RPE-EMT, which is consistent with EMT observed in hepatocellular or colorectal carcinoma cells in AIM2-deficient patients [42, 44]. Further examinations of whether genetic lesions of AIM2 render patients more susceptible to PVR following RD are required.

The present findings revealed that high levels of AIM2 expression play a crucial role in inhibiting the initial stage of RPE-EMT and maintaining homeostasis in the RPE in an inflammasome-independent manner. Additionally, high AIM2 expression levels are beneficial for maintaining homeostasis in the gastrointestinal tract [39]. In contrast, several studies have indicated that the activation of the AIM2 inflammasome can cause cell pyroptosis in central neurons [30, 36]. Based on the above observations, whether a high level of AIM2 expression is beneficial or damaging may depend on the specific cellular context. Consequently, before further use of AIM2 in a PVR setting, it is critical to establish precise signals that regulate AIM2 expression in the RPE and explore strategies to induce endogenous AIM2 expression to test whether its induction is protective or harmful to RPE-EMT and PVR progression. Alternatively, one therapeutic strategy involves decreasing *AIM2* expression, which leads to PVR progression and can be partially rescued by an AKT inhibitor, suggesting that suppression of AKT is a viable option for reducing PVR progression. Therefore, pharmacological targeting of AKT may be helpful in the treatment of individuals with AIM2-deficient PVR.

In summary, this study provides evidence that *AIM2*, typically recognized as a DNA sensor or gene related to cancer, plays a crucial role in RPE-EMT and proliferation, thereby contributing to the development of PVR in the eye. AIM2 exerts these effects by suppressing AKT activation in an inflammasome-independent manner. These findings provide a novel mechanistic explanation for abnormal RPE-EMT and proliferation in retinal diseases and identify potential therapeutic avenues for treating PVR.

## Supplementary information

Supplemental material 1

Supplemental material 2

Supplemental material 3

## Data Availability

The datasets generated during and/or analyzed during the current study are available from the corresponding author upon reasonable request.
