# Supplementary material for: Absent in melanoma 2: a potent suppressor of retinal pigment epithelial-mesenchymal transition and experimental proliferative vitreoretinopathy
Source: Cell Death Dis. 2025 Jan 27;16(1):49. doi: 10.1038/s41419-025-07367-9 (PMC11772762; doi:10.1038/s41419-025-07367-9)
Supplement: Supplementary file 1 — Supplemental material 1 [file 41419_2025_7367_MOESM1_ESM.docx]

# Supplemental information Supplemental Table 1

siRNA sequences

| siRNA | Sequence (5’-3’) |
| --- | --- |
| si-C | UUCUCCGAACGUGUCACGUTT |
| si-AIM2-1 | GAGAGUAAAUACAAGGAGA |
| si-AIM2-2 | CCCUUGGAACAAUUGUGAAUGGUUU |

Primer sequences for real-time PCR analysis

| Primers | Sequence (5’-3’) |
| --- | --- |
| *GAPDH*-F | GTGAAGGTCGGAGTCAACGG |
| *GAPDH*-R | CGGTGCCATGGAATTTGCC |
| *AIM2*-F | CGTGCTGCACCAAAAGTCTC |
| *AIM2*-R | GGCAAACAGCGCTTCTGAAA |
| *CDH1*-F | CACCCTGGCTTTGACGCCGA |
| *CDH1*-R | AAAATTCACTCTGCCCAGGACGCG |
| *CDH2*-F | CCTGTGGGAATCCGACGAATG |
| *CDH2*-R | GCCGCTTTAAGGCCCTCATTA |
| *VIM*-F | AGAGAACTTTGCCGTTGAAGC |
| *VIM*-R | ACGAAGGTGACGAGCCATT |
| *FN1*-F | AAGACCATACCCGCCGAATG |
| *FN1*-R | GGCATTTGGATTGAGTCCCG |
| *ACTA2*-F | CAGAAGGAGATCACGGCCCTAG |
| *ACTA2*-R | CGGCTTCATCGTATTCCTGTTTG |

**Supplemental figures**

**
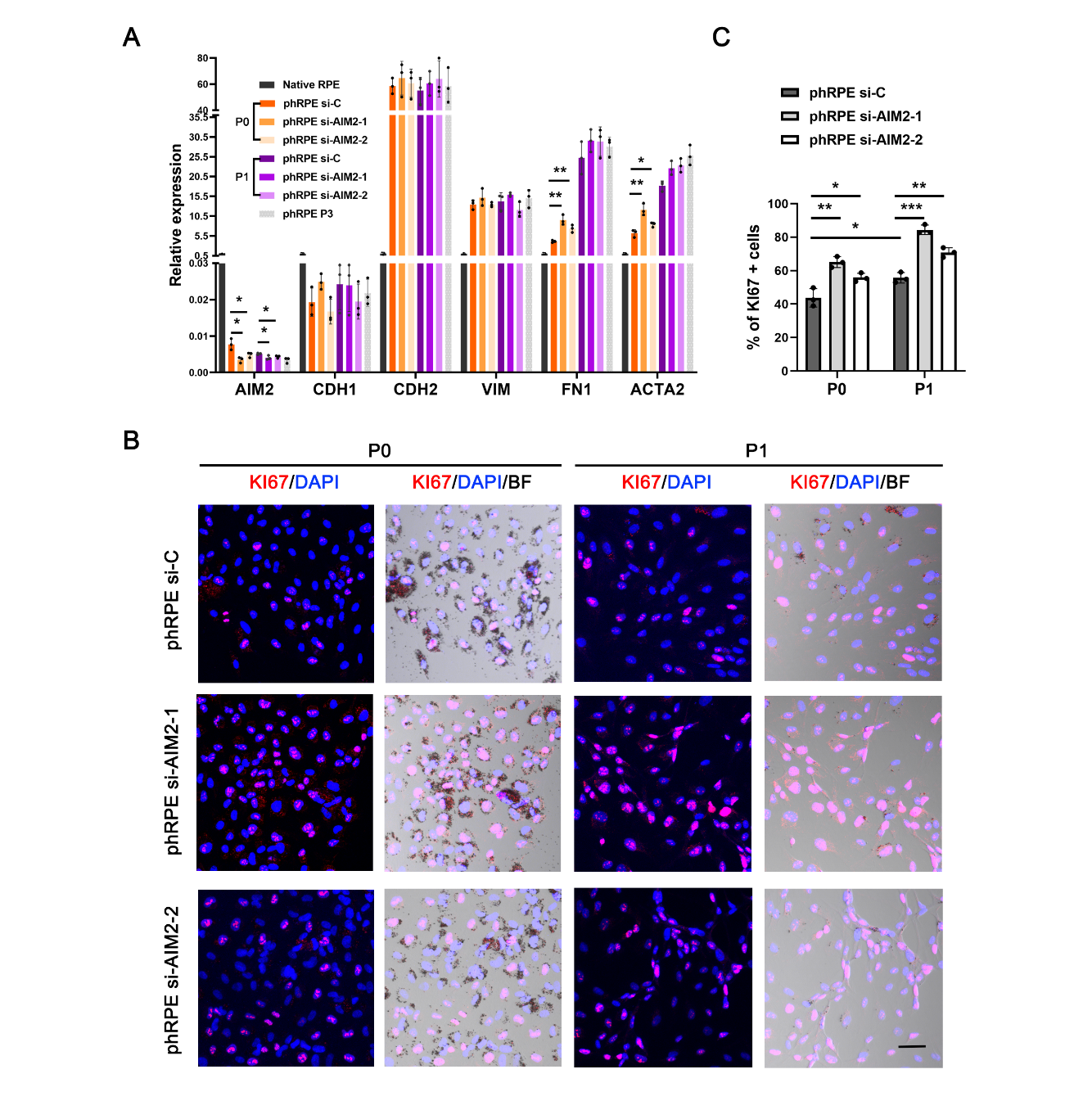
**

**Supplementary Fig. S1. Knockdown of AIM2 inhibits phRPE cell proliferation and EMT in cultures.** (A) phRPE cells were isolated from the eyes of donors. After 2 d in culture, phRPE cells were transfected with si-C, si-Aim2-1, or si-Aim2-2. Real-time PCR analysis of AIM2 and EMT-related genes was performed 5 d after transfection (P0, passage 0) or 10 d after transfection (P1, passage 1). N = 3. All data are presented as mean ± SD. **P* < 0.05, ***P* < 0.01 by one-way ANOVA and post hoc Bonferroni’s test. (B) Representative confocal IF images of KI67 in phRPE cells transfected with si-C, si-Aim2-1, or si-Aim2-2. Scale bar: 50 μm. (E) Quantifying the Ki67-positive cells based on the results (B) (n = 3). All data are presented as mean ± SD. **P* < 0.05, ***P* < 0.01, ****P* < 0.001 by one-way ANOVA and post hoc Bonferroni’s test.

**
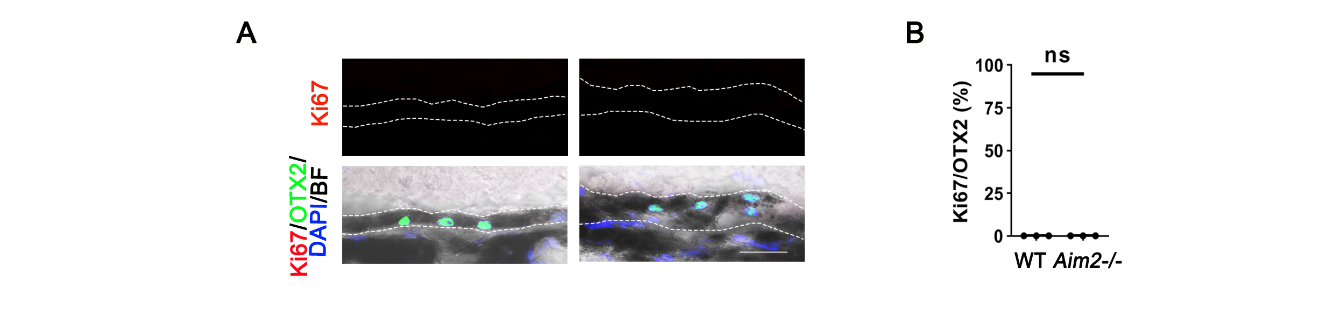
**

**Supplementary Fig. S2.** **AIM2 deficiency does not affect RPE proliferation in physiological conditions.** (A) Representative IF images of Ki67-positive (red) and OTX2-positive (green) in the RPE cryostat sections in 8-week-old WT and *Aim2*^−/−^ mice. Scale bar: 20 μm. (B) Quantifying the Ki67-positive cells/OTX2-positive cells based on the results (A). N = 3. ns: not significant by two-tailed Student’s t-test.

**
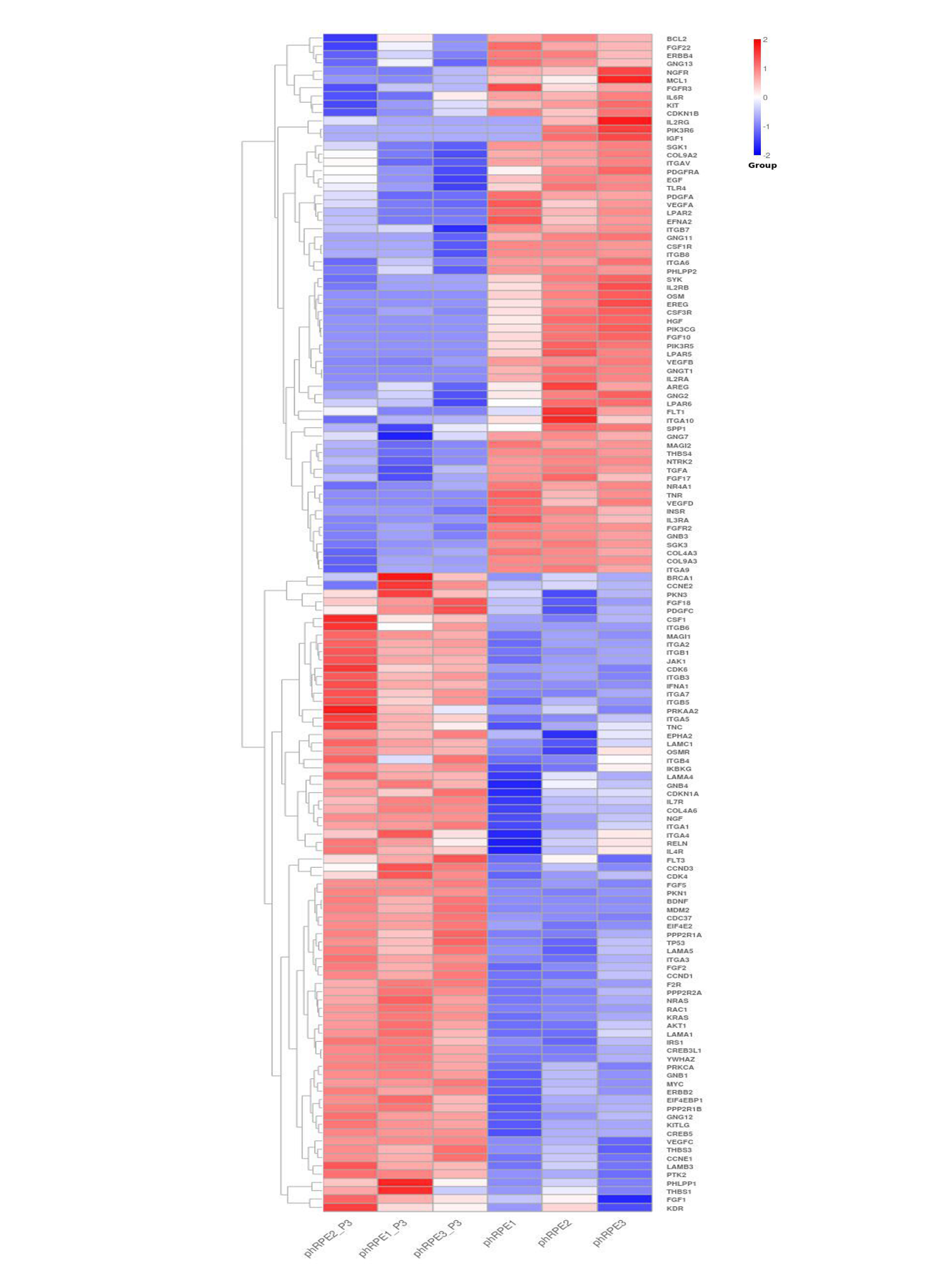
**

**Supplementary Fig. S3. A heatmap of PI3K-AKT signaling pathway from KEGG analysis of RNA-Seq in this study.** Heatmap represents a clustering of 142 DEGs within the PI3K-AKT signaling pathway based on KEGG analysis of RNA-Seq in native phRPE cells versus P3 phRPE cells.
