# Supplementary figures and images for: Absent in melanoma 2: a potent suppressor of retinal pigment epithelial-mesenchymal transition and experimental proliferative vitreoretinopathy
Source: Cell Death Dis. 2025 Jan 27;16(1):49. doi: 10.1038/s41419-025-07367-9 (PMC11772762; doi:10.1038/s41419-025-07367-9)

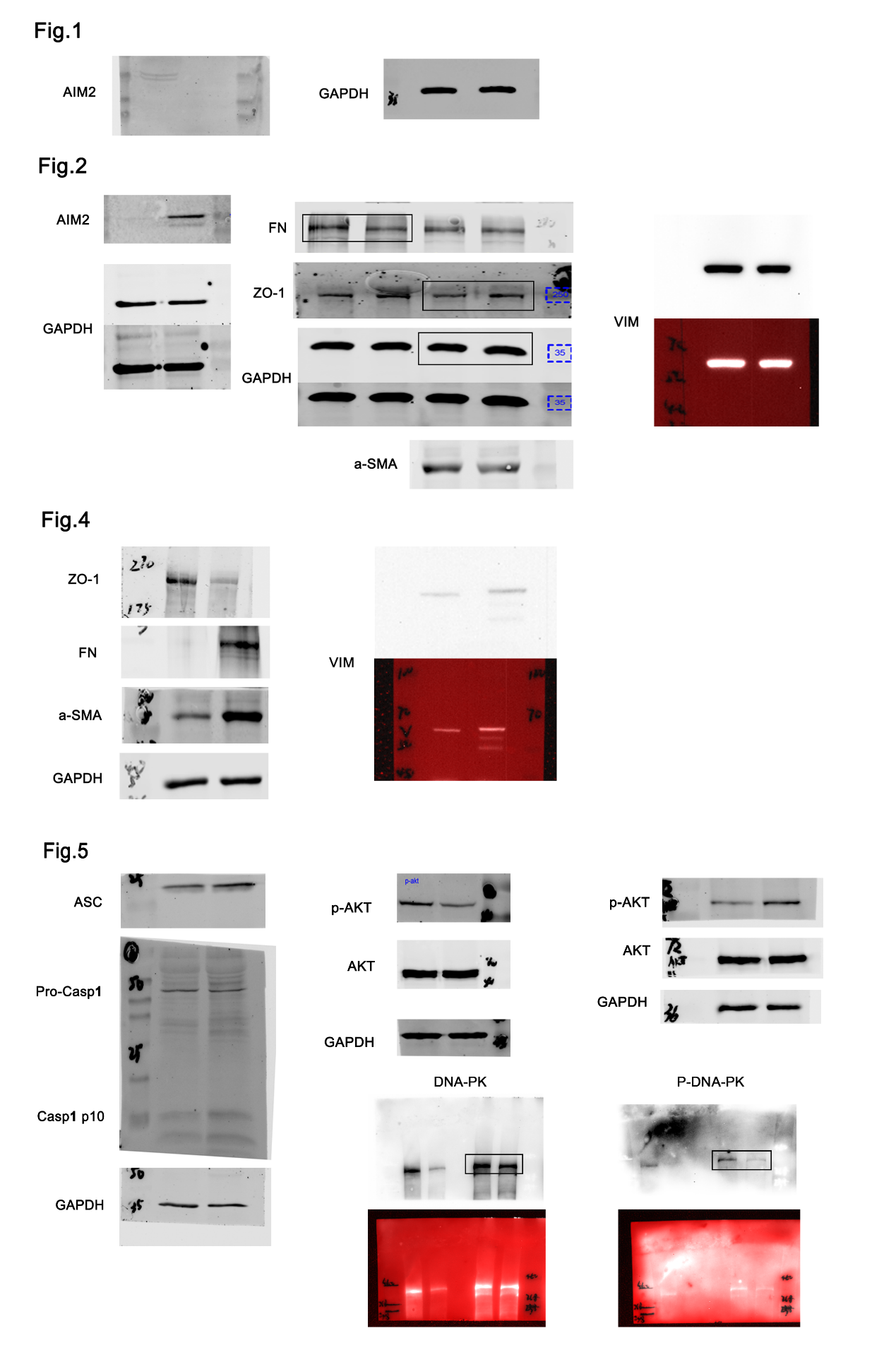


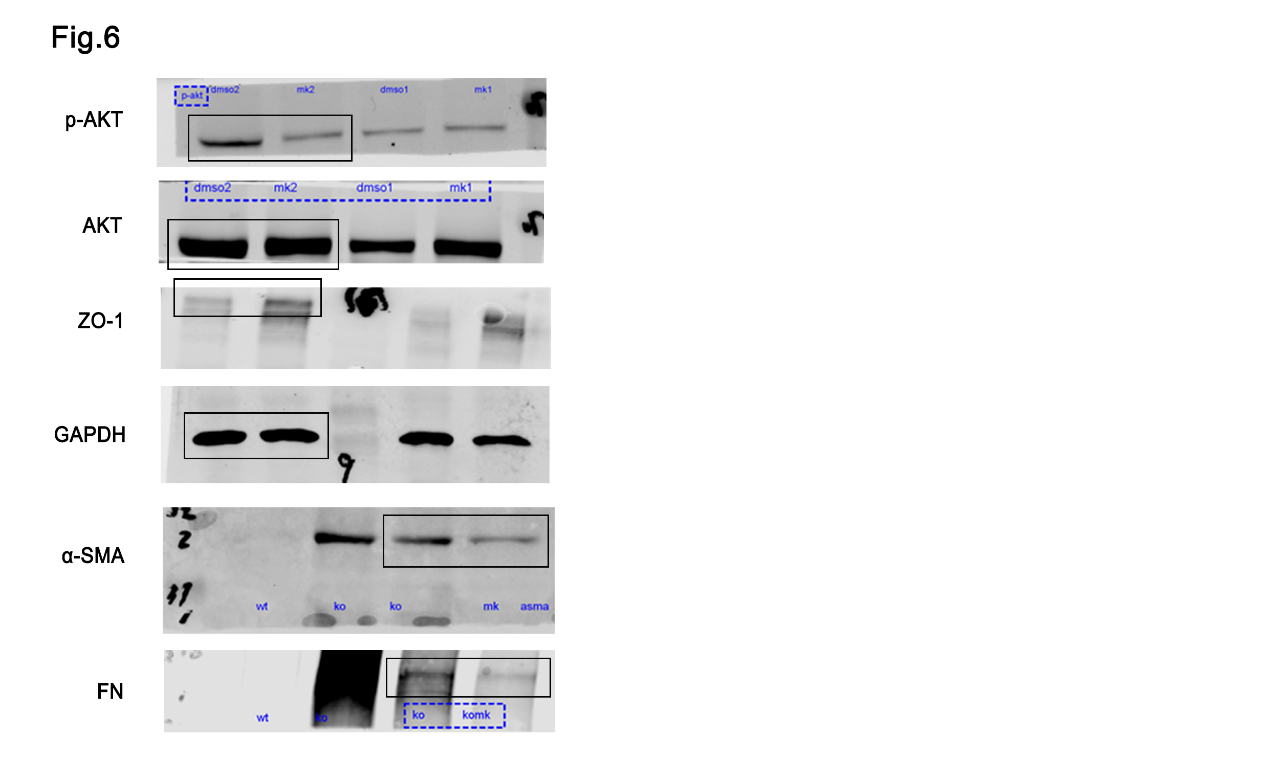

Supplement: Supplementary file 3 — Supplemental material 3 [file 41419_2025_7367_MOESM3_ESM.docx]
